# Immuncheckpointinhibitoren in der Behandlung der progressiven multifokalen Leukenzephalopathie

**DOI:** 10.1007/s00115-021-01194-x

**Published:** 2021-09-29

**Authors:** L. Nitsch, V. Kaps, V. Zschernack, N. Gancarczyk, F. van Essen, C. Schmeel, T. Klockgether, J. Zimmermann, M. Müller

**Affiliations:** 1 Klinik und Poliklinik für Neurologie, Universitätsklinikum Bonn, Venusberg-Campus 1, 53127 Bonn, Deutschland; 2 Klinik für Kinder- und Jugendmedizin, Universitätsmedizin Göttingen, Robert-Koch-Straße 40, 37075 Göttingen, Deutschland; 3 Institut für Neuropathologie, Universitätsklinikum Bonn, Venusberg-Campus 1, 53127 Bonn, Deutschland; 4 Klinik für Neuroradiologie, Universitätsklinikum Bonn, Venusberg-Campus 1, 53127 Bonn, Deutschland; 5 Deutsches Zentrum für Neurodegenerative Erkrankungen (DZNE), Venusberg-Campus 1, 53127 Bonn, Deutschland

## Kasuistiken

Die progressive multifokale Leukenzephalopathie (PML) ist eine meist verheerend verlaufende ZNS-Infektion, die durch das John-Cunningham(JC)-Virus verursacht wird. Die hier dargestellte Fallserie beschreibt die unterschiedlichen Verläufe einer PML und die Möglichkeit, Immuncheckpointinhibitoren in der Behandlung der PML einzusetzen.

## Fall 1

Es handelte sich um einen, damals 60-jährigen, Patienten mit 2018 diagnostiziertem follikulärem Lymphom. Nach 6 Zyklen Chemotherapie mit Bendamustin, dem monoklonalen Anti-CD20-Antikörper Obinutuzumab und anschließender Erhaltungstherapie mit Obinutuzumab 2‑monatlich (zuletzt 04/2020 erhalten) kam es zu einer stabilen Remission.

**Figure 1:**
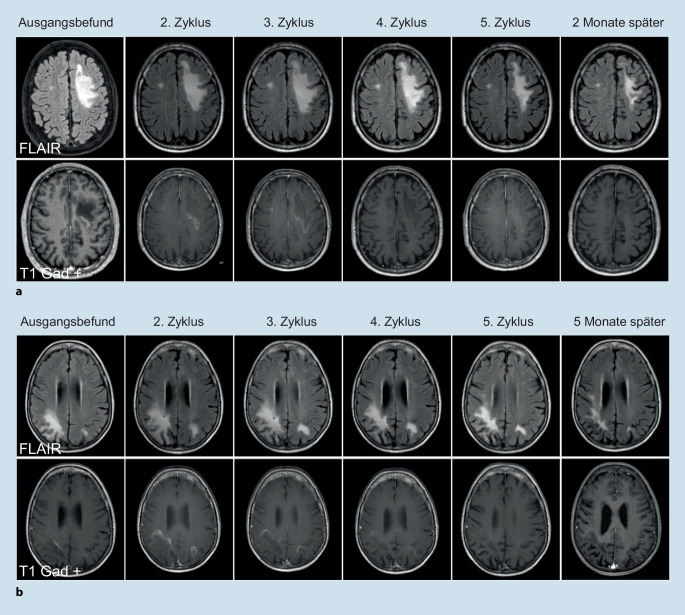

In 05/2020 traten eine Aphasie und eine rechtsseitige Armparese auf. In der zerebralen Magnetresonanztomographie (cMRT) zeigte sich eine links frontale, T2/fluid-attenuated-inversion-recovery(FLAIR)-hyperintense Läsion mit randständiger Kontrastmittelanreicherung sowie eine kleinere rechts frontale Läsion (Abb. 1a). Im peripheren Immunstatus waren CD19+-B-Zellen supprimiert (0/μl). Im Liquor waren die Routineparameter unauffällig, aber mittels Polymerase-Kettenreaktion (PCR) gelang der Nachweis von JC-Polyomavirus-DNA, sodass die Diagnose einer PML gestellt wurde. Unter der initialen Behandlung mit Mirtazapin und Mefloquin kam es klinisch und bildgebend zu einer weiteren Verschlechterung.

Nach Übernahme in unsere Klinik begannen wir eine Therapie mit dem Immuncheckpointinhibitor Pembrolizumab (2 mg/kg Körpergewicht) in 4‑wöchentlichen Abständen. Zu Beginn des 3. Zyklus kam es zu einer leichten Zunahme der Aphasie und korrelierenden Größenprogredienz der Läsion mit weiterhin randständiger Kontrastmittelanreicherung in der MRT-Bildgebung (Abb. 1a). Nach 2 weiteren Zyklen verbesserte sich jedoch die Armparese. Die FLAIR-hyperintensen Marklagerveränderungen und die randständige Kontrastmittelanreicherung waren regredient. Passend dazu war die Kopienzahl der JC-Virus-DNA im Liquor rückläufig und zu Beginn des 4. Zyklus nicht mehr nachweisbar.

Wir beendeten die Therapie damit nach insgesamt fünf Zyklen. In einem Verlaufs-cMRT zwei Monate nach Abschluss der letzten Pembrolizumabinfusion kam es zu einer weiteren Rückbildung der Läsion. Eine Schrankenstörung war nicht mehr nachweisbar. Klinisch kam es ebenfalls zu einer Stabilisierung.

## Fall 2

Die damals 76-jährige Patientin wurde von 2016 bis 2019 aufgrund eines splenischen Marginalzonenlymphoms mit insgesamt 6 Zyklen Bendamustin und Rituximab behandelt. Darunter kam es zu einer stabilen Remission. 05/2020 traten eine Hemiparese links, eine homonyme Hemianopsie nach links und epileptische Anfälle auf. Im cMRT zeigte sich eine T2/FLAIR-hyperintense Läsion rechts parietal mit Ausdehnung bis nach okzipital unter Aussparung des Kortex ohne Kontrastmittelanreicherung (cMRT nicht gezeigt). Es lag eine Leukopenie mit 1,6 G/l und eine Suppression von CD19+-B-Zellen (0/μl) vor. Die Routineparameter im Liquor waren wie im Fall zuvor unauffällig, oligoklonale Banden waren nicht nachweisbar. JC-Polyomavirus-DNA war wiederholt nicht detektierbar, die weitere Erregerdiagnostik blieb ebenfalls unauffällig. Anti-JC-Virus-Antikörper waren im Serum nicht vorhanden. Es erfolgte eine Verlaufsuntersuchung nach 4 Wochen mit cMRT-bildgebend progredienten bis nach subkortikal reichenden T2/FLAIR-Läsionen rechts parietotemporal sowie geringer auch links parietal mit einer neu aufgetretenen Schrankenstörung (Abb. 1b). Es wurde daraufhin eine zerebrale Biopsie durchgeführt, die histologisch die Diagnose einer PML ergab. Es erfolgten 5 Therapiezyklen mit Pembrolizumab (2 mg/kg Körpergewicht) in 4‑wöchentlichen Abständen. Während es zu Beginn des 2. Zyklus zunächst im cMRT zu einer Zunahme der randständigen Kontrastmittelanreicherung kam, zeigten sich im weiteren Verlauf die T2/FLAIR-hyperintensen Läsion sowie die randständige Schrankenstörung rückläufig. Neue Beschwerden traten nach Abschluss der Therapie nicht auf. Die Feinmotorik und Koordination verbesserten sich.

## Fall 3

2014 erfolgten bei der damals 53-jährigen Patientin aufgrund eines nichtkleinzelligen pulmonalen Adenokarzinoms eine Resektion und adjuvant eine Radiochemotherapie mit Vinorelbin und Carboplatin. Als weitere relevante Vorerkrankung bestand eine seronegative Arthritis mit Z. n. Sulfasalazin‑, Prednisolon‑, Resochin‑, Methotrexatbehandlung sowie bei Vorstellung Einnahme von Leflunomid.

**Figure 2:**
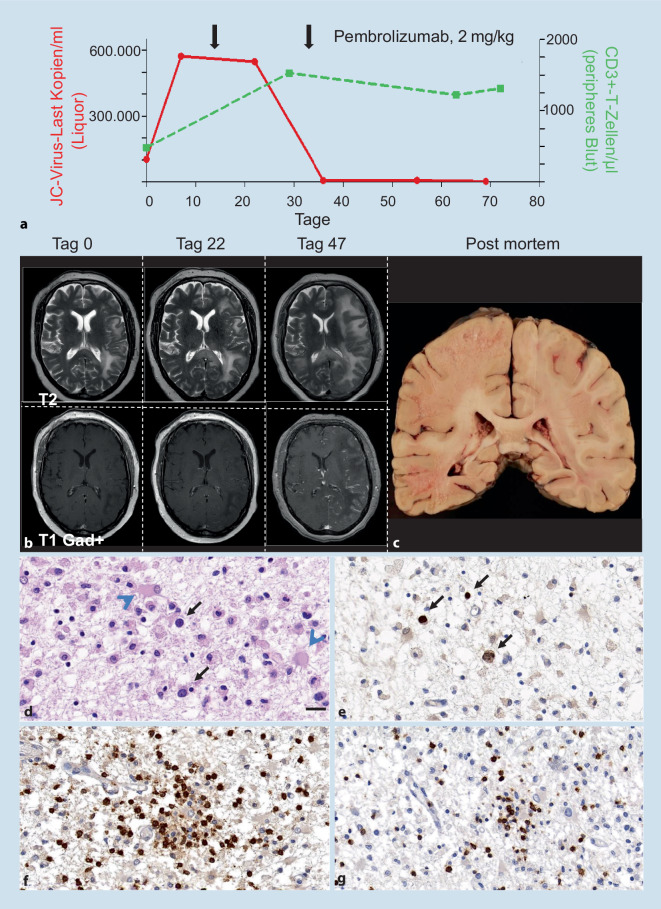

2017 kam es zu über Monate langsam progredienten Wortfindungsstörungen und einer leichtgradigen Hemiparese rechts. In einem cMRT zeigte sich eine frontoparietale Läsion links. Im Liquor waren die Routineparameter regelrecht, aber JC-Virus-DNA war mittels PCR nachweisbar. Die Kopienzahl betrug initial 103.000 Kopien/ml und stieg 9 Tage später auf 570.000 Kopien/ml an. Bei klinisch und bildgebender Progredienz unter einer extern begonnenen Therapie mit Mirtazapin, Mefloquin sowie Elimination des Leflunomids mittels Colestyramin begannen wir daraufhin eine Pembrolizumabtherapie (2 mg/kg Körpergewicht) und verabreichten nach 3 Wochen die zweite Infusion. Zehn Tage nach der ersten Infusion sank die JC-Virus-Last und fiel nach der zweiten Infusion weiter, sodass die ursprüngliche Viruslast 5 Wochen nach der zweiten Infusion um 99,5 % gesunken war (Abb. 2a). Zusätzlich stieg die CD3+-T-Zell-Zahl im peripheren Blut von 481 T-Zellen/μl vor Beginn mit Pembrolizumab auf 1310 T‑Zellen/μl an (Abb. 2a). Bildgebend zeigte sich jedoch nach dem zweiten Behandlungszyklus ein bihemisphärischer Progress der T2/FLAIR-hyperintensen Läsionen mit ödematöser Schwellung und flauer Kontrastmittelanreicherung (Abb. 2b). Wir leiteten eine Kortikosteroidbehandlung bei V. a. ein Immunrekonstitutionssyndrom (IRIS) ein, dennoch verschlechterte sich der klinische Zustand weiter. Aufgrund von rezidivierenden Pneumonien und epileptischen Anfällen erfolgte eine intensivmedizinische Behandlung über mehrere Wochen. Im Verlauf erfolgte die Entscheidung für eine palliative Behandlung entsprechend dem mutmaßlichen Patientenwillen und die Patientin verstarb nach kurzer Zeit.

Postmortal konnten wir makroskopisch großflächige Nekrosen linkshemisphärisch und eine beginnende Demyelinisierung rechtshemisphärisch feststellen (Abb. 2c). Histologisch zeigten sich in der Hämatoxylin-Eosin(HE)-Färbung der betroffenen Bereiche abnorme Astrozyten und vergrößerte Oligodendrozyten (Abb. 2d) mit immunhistochemischem Nachweis des Polyomavirus-T-Antigens (Abb. 2e). Darüber hinaus konnten wir eine massive Infiltration von CD3+-T-Zellen (Abb. 2f) und hierunter auch Programmed-cell-death-1(PD-1)-positive Zellen (Abb. 2g) nachweisen.

## Diskussion

JC-Virus Infektionen führen zu einer latenten Infektion im Uroepithelium der Niere und lymphatischen Gewebe [1]. In serologischen Studien ergibt sich eine Prävalenz einer erfolgten JC-Virus-Infektion von 60 bis 80 % bei unter 70-Jährigen [4]. Wenn die Immunantwort durch Erkrankungen oder eine iatrogene Immunsuppression wie z. B. Natalizumab oder eine Chemotherapie reduziert wird, kann die latente JC-Virus-Infektion zu einer PML führen. Die PML ist eine meist verheerend verlaufende ZNS-Infektion. Sämtliche direkt antiviral ausgerichteten Therapiestudien verliefen bislang negativ, die Prognose hängt vor allem von der Rekonstitution des Immunsystems ab. Bleibende Defizite und fulminante Verläufe sind häufig. Die hier vorgestellten Fälle verdeutlichen die unterschiedlichen Verläufe einer PML mit zum Teil schwieriger Diagnosefindung (Fall 2) oder hochakutem Verlauf (Fall 3). In Fall 1 und 2 hatten die Patienten neben Bendamustin eine CD20-depletierende Antikörpertherapie erhalten sowie in Fall 3 eine Behandlung mit Leflunomid bei Z. n. Chemotherapie mit Vinorelbin und Carboplatin. Zu beachten ist dabei, dass CD20-depletierende Antikörpertherapien sowie der Leflunomidmetabolit Teriflunomid auch in der Neurologie zur Behandlung von Multiple-Sklerose-Patienten eingesetzt werden. Während und nach einer immunmodulatorischen Behandlung, insbesondere bei zusätzlichen Vortherapien, sollte daher immer die Wachsamkeit bzgl. der Entwicklung einer PML bestehen.

Behandlungsansätze zur Therapie der PML beinhalten Substanzen, deren Wirksamkeit mehrheitlich aus In-vitro-Daten abgeleitet wurden: Hierzu gehören Mirtazapin, das als Antagonist des 5‑HT_2A_-Rezeptors den viralen Eintritt in Gliazellen hemmt, oder das Antimalariamedikament Mefloquin. Der Effekt ist in der klinischen Anwendung jedoch sehr begrenzt [2, 5], sodass die Rekonstitution des Immunsystems eine zentrale Rolle einnimmt.

Wenn, wie in unseren genannten Fällen, das Wiederherstellen des Immunsystems z. B. durch Beenden einer immunsuppressiven Therapie, Beginn einer hochaktiven antiretroviralen Therapie zur Behandlung einer HIV-Infektion oder Elimination von Natalizumab durch Plasmapherese keinen ausreichenden Behandlungsansatz darstellt, ist die Gabe von Immuncheckpointinhibitoren eine mögliche Therapieoption.

In den letzten Jahren wurden positive Fallberichte und -serien zur Behandlung der therapierefraktären PML mit Immuncheckpointinhibitoren veröffentlicht [3]. Die Immuncheckpointinhibitoren Pembrolizumab und Nivolumab binden an den PD-1-Rezeptor von T‑Zellen und inhibieren damit die Interaktion zwischen PD‑1 und dessen Liganden PD-L1 und PD-L2, welche ansonsten zu einer Suppression der zellulären Immunantwort führen würde. Dieses Therapieprinzip wird seit mehreren Jahren zur Therapie unterschiedlicher, fortgeschrittener Tumorerkrankungen eingesetzt. PD‑1 und seine Liganden sind jedoch auch in chronischen viralen Entzündungen wie der PML heraufreguliert und das JC-Virus nutzt damit diesen Immun-Escape-Mechanismus [9, 11].

Der Therapieeffekt von Immuncheckpointinhibitoren bei PML ist bislang nicht in randomisierten klinischen Studien untersucht. Veröffentlichte Fallberichte und -serien beschreiben ein mögliches klinisches und bildgebendes Ansprechen unter der Therapie [3, 7, 10]. Auch in allen drei hier beschriebenen Fällen lässt sich ein deutlicher Abfall der Viruslast oder bildgebendes Ansprechen auf die Immuncheckpointinhibition nachweisen, in den ersten beiden Fällen zeigt sich auch klinisch eine Stabilisierung unter der Therapie. Der klinische Ausgang ist jedoch heterogen und sollte in unabhängigen klinischen Studien überprüft werden, da nach initialer Stabilisierung ein Rezidiv auftreten kann, die PML weiter voranschreiten kann oder die bereits eingetretene Hirnschädigung so ausgeprägt sein kann, dass, wie in unserem dritten Fall, ein Fortführen der Therapie nicht vielversprechend ist [3, 6, 8].

Ein bekanntes Phänomen der PML ist nach eingeleiteter Immunrekonstitution das Auftreten eines PML-IRIS, welches zu einer sekundären klinischen Verschlechterung führen kann. Der bildgebend etablierte Nachweis eines PML-IRIS ist das Auftreten einer Kontrastmittelaufnahme an den Rändern der PML-Läsion sowie die Entwicklung eines raumfordernden Ödems [1]. In einer solchen Situation ist es wichtig, rechtzeitig Steroide zu geben, um eine Schädigung durch die Raumforderung zu vermeiden [1]. Alle drei Fälle zeigten bildgebend Zeichen eines PML-IRIS, wohingegen ein raumfordernder Effekt nur im letzten Fall auftrat. So wurde in den ersten beiden Fällen auf eine erneute Abschwächung der Immunrekonstitution durch Steroide verzichtet. Das PML-IRIS des dritten Falls ließ sich auch postmortal histologisch bestätigen: hier zeigte sich eine massive Entzündungsreaktion mit infiltrierenden CD3+-T-Zellen in JC-Virus-infizierte Areale, ein PML-IRIS-typischer Befund. Gleichzeitig belegte die ansteigende Zahl der CD3+-T-Zellen im peripheren Blut sowie der Abfall der JC-Virus-Last im Liquor das Therapieansprechen auf Pembrolizumab. Dieser Fall belegt eindrücklich, dass ein früher Therapiebeginn erforderlich ist, damit Patienten mit einer PML von einer solchen Therapie auch klinisch profitieren können.

Zusammenfassend verdeutlicht die Fallserie die komplizierten und schweren Verläufe einer PML und betont die Wichtigkeit neuer Behandlungsansätze wie der Anwendung von Immuncheckpointinhibitoren.

## Fazit für die Praxis

- Bei neurologischen Defiziten und passender zerebraler Bildgebung mit flächigen, subkortikalen Läsionen sowie zurückliegender oder bestehender Immunsuppression sollte an eine progressive multifokale Leukenzephalopathie (PML) gedacht werden.
- Auch bei negativem John-Cunningham(JC)-Virus-DNA-Nachweis im Liquor kann eine PML vorliegen.
- Kontrollierte klinische Studien zum Einsatz von Checkpointinhibitoren zur Behandlung der PML stehen zurzeit noch aus.
- Eine Therapie mit Immuncheckpointinhibitoren als individueller Heilversuch ist eine Therapieoption bei einer PML, wobei der frühzeitige Therapiebeginn für eine erfolgreiche Therapie entscheidend ist.
